# Editorial: Psychiatric Comorbidities in the Epilepsies: Extensive Mechanisms and Broad Questions

**DOI:** 10.3389/fnint.2022.951170

**Published:** 2022-06-17

**Authors:** Rafael Naime Ruggiero, Jose Eduardo Peixoto-Santos, Lezio Soares Bueno-Junior, Kette D. Valente, Joao Pereira Leite

**Affiliations:** ^1^Department of Neurosciences and Behavioral Sciences, Ribeirao Preto Medical School, University of São Paulo (USP), Ribeirao Preto, Brazil; ^2^Neuroscience Sector, Department of Neurology and Neurosurgery, Universidade Federal de São Paulo (UNIFESP), São Paulo, Brazil; ^3^Department of Psychiatry, University of Michigan Medical School, Ann Arbor, MI, United States; ^4^Institute and Department of Psychiatry, Faculty of Medicine of the University of São Paulo (HCFMUSP), São Paulo, Brazil

**Keywords:** epilepsy, depression, psychosis, suicidality, anxiety, pharmacoresistance

Epilepsy is one of the most prevalent neurological diseases, affecting almost 70 million people globally and causing a significant burden to patients, their families, and healthcare systems (O'Donohoe et al., 2020). Neurodevelopmental delay during childhood, socioeconomic issues in adulthood, and comorbidities across age groups worsen the burden of seizures (Kanner, 2009; Ruggiero et al., 2012; Laxer et al., 2014; Chatterjee et al., 2020; Kobow et al., 2020; Oliveira et al., 2022). Psychiatric comorbidities affect persons with epilepsy regardless of seizure control. For instance, a Canadian-based study showed a 35% lifetime prevalence of mental health in persons with epilepsy compared to 20.7% in the average population (Tellez-Zenteno et al., 2007).

This Research Topic gathers experimental and clinical articles devoted to the elusive relationship between epilepsies and psychiatric disorders, ranging from molecular biology to treatment options. It is no surprise that most articles address research on mesial temporal lobe epilepsy (TLE). Psychiatric disorders, namely depression, are more likely to occur in mesial TLE than in neocortical TLE or extratemporal epilepsies (Dalmagro et al., 2012). This strong link between mesial TLE and psychiatric comorbidities is seen as an epiphenomenon since limbic structural abnormalities that characterize mesial TLE may play a vital role in mood disorders (Kandratavicius et al., 2012b,c; Valente and Busatto Filho, 2013).

Michaelis et al. described the frequency of mood disorders, anxiety disorders, and functional seizures at a tertiary health care center. Their study reinforced the need for psychiatric screening in persons with epilepsy. Diagnosing these comorbidities with higher precision is crucial for the proper management of patients, corroborating previous studies (Kanner, 2016; Ribot et al., 2017; Michaelis et al., 2018; Asadi-Pooya et al., 2019; Baroni et al., 2021; Jungilligens et al., 2021).

Kanner addressed the prevalence, risk factors, and shared mechanisms of suicidality in persons with epilepsy. The author reviewed how different psychiatric comorbidities, seizures, and genetic and social factors impact suicidality. Kanner also emphasizes the relevance of screening in preventing suicide in these patients. As other studies have shown several risk factors for suicidality in persons with epilepsy (Jones et al., 2003; Mula et al., 2013; Pugh et al., 2013), this review by Kanner could be of broad interest. Kanner and Michaelis et al. clinical papers emphasize the urgent need for integrated mental health care to improve quality of life and decrease mortality in persons with epilepsy.

Bandeira et al. described changes in the methylation status of BDNF and SLC6A4 promoters in peripheral blood samples from persons with TLE with mood and anxiety disorders. The authors also analyzed other potential factors associated with TLE comorbidities, including genes widely explored in psychiatry and epileptology (Hu and Russek, 2008; Esmail et al., 2015), and could shed light on specific differences between types of psychiatric comorbidity. Methylation studies are advancing our comprehension of psychiatric and neurologic diseases in general (Bakusic et al., 2017; Capper et al., 2018; Barbu et al., 2022; Jabari et al., 2022).

Visoná de Figueiredo et al. investigated associations between different types of hippocampal sclerosis (as defined by the International League Against Epilepsy in Blümcke et al., 2013) and depression. They also studied whether neuron density differs between TLE patients with and without depression. Their study follows up on previous reports on histopathological differences between patients with and without psychiatric comorbidities in general (Kandratavicius et al., 2012a, 2014, 2015) and between types of hippocampal sclerosis and epilepsy-related factors (Coras et al., 2014; Thom, 2014; Rodrigues et al., 2015; Dombroski et al., 2020).

Godoy et al. reviewed studies on parvalbumin-positive interneuron populations in patients and animal models of epilepsy. They also described the GABAergic contribution to psychiatric comorbidities and how treatments could impact the function of these interneurons. Godoy et al. contributed a timely review, as the role of these interneurons in epilepsy and psychiatric diseases are increasingly gaining recognition (Jiang et al., 2016; Taylor et al., 2019; Spijker et al., 2020).

Bragatti reviewed the history and possible mechanisms of forced normalization. This phenomenon, where psychotic-like behavior emerges in some TLE patients after successful seizure control, is still not fully understood. Bragatti reinforced the controversies around forced normalization and discussed theories to explain the mismatch between animal models and human data. As psychosis adds a vital burden to persons with epilepsy (Bragatti et al., 2010; de Toffol et al., 2018), a better understanding of forced normalization vs. epilepsy-unrelated psychosis may improve patient care.

Mota et al. investigated whether a self-applied transcranial direct-current stimulation (tDCS) over the dorsolateral prefrontal cortex was better than a placebo at treating depression and anxiety in TLE patients. Their results are interesting and reinforce the need for further investigation, especially when compared to studies showing the efficacy of tDCS in depressive patients without epilepsy (Zhang et al., 2021). Non-pharmacological treatments like tDCS are promising at reducing both the adverse effects and the number of medications, two critical factors behind the low adherence to pharmacological treatment in TLE (Laville et al., 2018; Steinert and Fröscher, 2018).

In summary, this Research Topic is of interest to epileptologists, psychiatrists, and laboratory researchers. Although informed health care professionals recognize the psychiatric aspects of epilepsy, managing the affected patients is still challenging (Gandy et al., 2021), which continues to justify basic and clinical research.

## Author Contributions

All authors listed have made an equally substantial, direct, and intellectual contribution to the work and approved it for publication.

## Funding

We thank Fundação de Amparo á Pesquisa do Estado de São Paulo (FAPESP, grants #16/17882-4 and #18/02303-4) and Conselho Nacional de Desenvolvimento Científico e Tecnológico (CNPq, grants #422911/2021-6 and #307817/2019-9) for the financial support.

## Conflict of Interest

The authors declare that the research was conducted in the absence of any commercial or financial relationships that could be construed as a potential conflict of interest.

## Publisher's Note

All claims expressed in this article are solely those of the authors and do not necessarily represent those of their affiliated organizations, or those of the publisher, the editors and the reviewers. Any product that may be evaluated in this article, or claim that may be made by its manufacturer, is not guaranteed or endorsed by the publisher.
